# Multi-Segment Leads To Reduce RF Heating in MRI: A Computational Evaluation at 1.5T and 3T

**DOI:** 10.1109/EMBC40787.2023.10340219

**Published:** 2023-07

**Authors:** Tayeb Zaidi, Giorgio Bonmassar, Laleh Golestanirad

**Affiliations:** 1 Department of Biomedical Engineering, Northwestern University, McCormick School of Engineering, Evanston IL and Department of Radiology, Feinberg School of Medicine, Northwestern University, Chicago IL.; 2 Martinos Center for Biomedical Imaging, Mass General Research Institute, Boston MA and Department of Radiology, Harvard Medical School, Cambridge MA

## Abstract

Implanted neurostimulators are currently in widespread use and allow patients to receive therapeutic nerve stimulation for a variety of conditions. Such devices often make use of long leads extending from the device to the relevant nerve to deliver their stimulation. These leads carry a significant radiofrequency (RF) safety concern for patients who also receive magnetic resonance imaging (MRI) scans. The incident RF energy from the MRI body coil can couple with the lead and produce dangerous levels of heating at the tip of the lead during a scan. Recent studies have shown one useful approach to mitigate this heating is to vary the conductivity of the wire along its length to decrease the coupling of the incoming RF energy from the MRI coil with the long lead. In this study, we adopt a similar approach and extend it by segmenting a long cylindrical lead model into two sections of differing conductivities and assessing the maximum 1g specific absorption rate (SAR) at the lead tip at both 64 MHz and 127 MHz. We also evaluated the effect of insulation thickness as well as conductivity of the phantom on the maximum 1g SAR. An 11-fold reduction in the SAR was achieved when using high conductivity ratios between the two wire segments for the 127 MHz coil and a 2-fold reduction was seen for the 64 MHz coil.

## I. Introduction

Active implantable medical devices (AIMDs), such as deep brain stimulation (DBS) devices, peripheral nerve stimulators, and pacemakers, provide electrical stimulation to nerves throughout the body for therapeutic purposes. Patients with these devices often require magnetic resonance imaging (MRI) for follow-up [1], to assess the effectiveness of stimulation [2], and for general clinical needs.

During MRI, radiofrequency (RF) induced heating can occur when the electric field from the MRI’s RF coil interacts with an implanted metallic lead within the patient. If the absorbed energy is high, dangerous levels of heating can cause permanent tissue damage [3], [4], [5]. RF induced heating typically occurs near the exposed tip of long leads and can be quantified by the specific absorption rate (SAR). To mitigate this potential safety risk, manufacturers have established strict guidelines for MRI protocols. These procedures are typically conducted using a 1.5 T horizontal closed-bore scanner and must adhere to established heating limits such as $B1_{RMS}^{+}<1.1\;\mu T$, or whole-head SAR <0.1 W/kg [6]. Such guidelines limit clinical MRI for patients with AIMDs.

One approach to reduce RF heating is to implement a modified lead design that varies the conductivity along its length [7], [8]. Changing the conductivity sharply at a point along the lead causes reflections that generate more heterogeneous heating throughout the lead as opposed to at the tip. This approach has been tested and validated through simulations and in experiments with a two-segment stripline lead model. A nearly 2-fold SAR reduction was achieved when testing the resistive tapered stripline (RTS) at 127 MHz.

In this study, we assess the potential SAR reduction of a two-segment cylindrical lead model at 64 MHz and 127 MHz for various conductivity ratios. We also examine how lead insulation thickness and conductivity of the surrounding tissue impact SAR reduction for different lengths of the two lead segments.

## II. Methods

### A. RF Coil and Phantom Design

Simulations were performed with models of two RF transmit coils, implemented in ANSYS Electronics Desktop 2021 R1 (ANSYS Inc., Canonsburg, PA) HFSS. The coils were 16-leg high pass birdcage coils, each tuned to its primary resonant modes at either 64 MHz and 127 MHz and excited to produce circularly polarized magnetic fields. To ensure consistency, the input power of both coils was adjusted to achieve a mean B1^+^ value of 3μT within a 10 mm axial plane at the center of each coil.

**Fig. 1.**
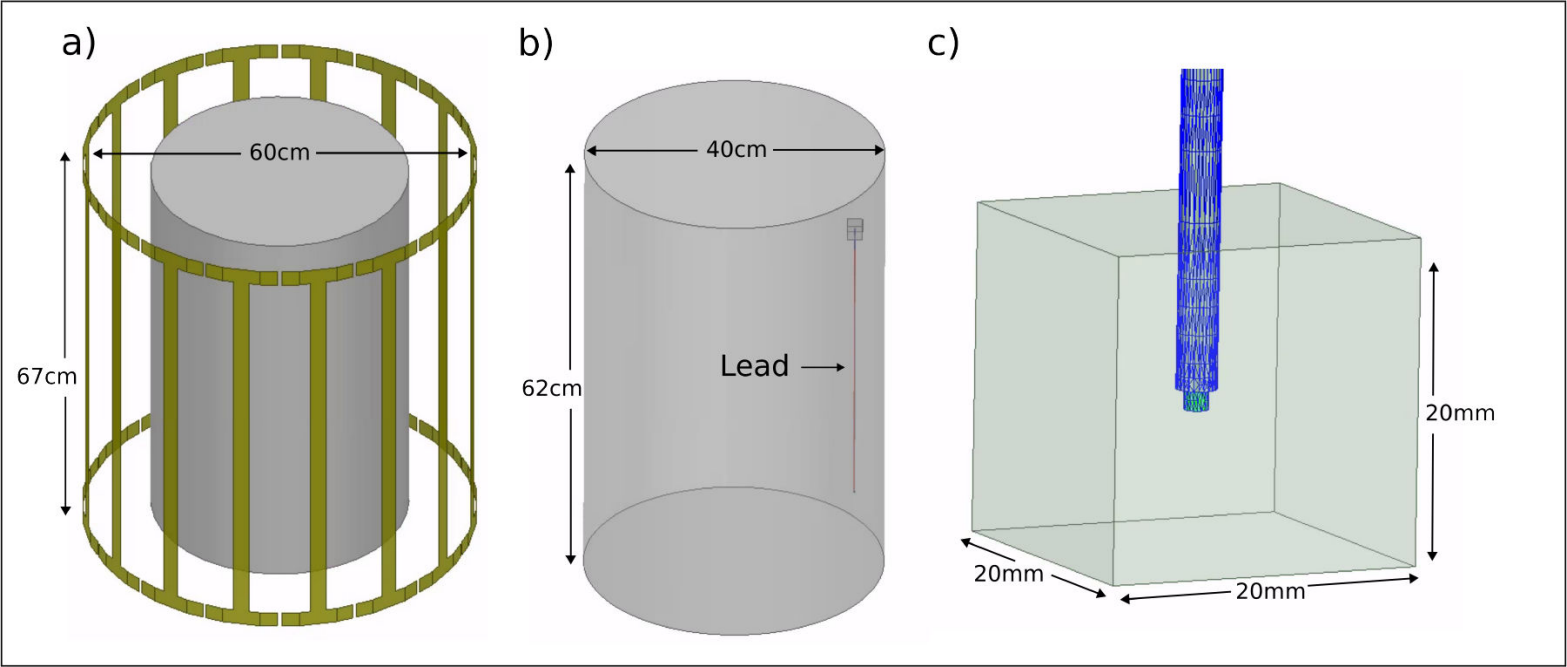
(a): The RF birdcage coil can be seen with the phantom located at the isocenter of the coil. (b): The phantom shown with the implant present on the right-hand side. The small box at the tip of the implant is the region where the maximum SAR was measured. (c): Zoomed in diagram of the SAR box surrounding the meshed lead tip.

A cylindrical phantom, with dimensions of 200 mm in radius and 620 mm in height, was used in simulations. The properties of the phantom, including its conductivity (0.47 S/m) and relative permittivity (80) were set to match the ASTM F2182 standard for RF heating tests [9]. The phantom was positioned at the center of both coils as depicted in Figure 1.

### B. Cylindrical Segmented Lead

**Fig. 2.**
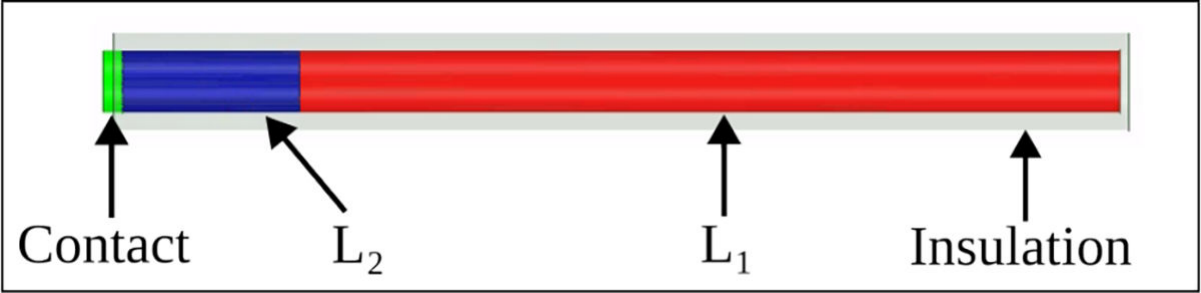
Side view of the cylindrical lead. Conductor 2 (blue) and conductor 1 (red) are surrounded by the insulation (transparent). Wire diameter was set to 1.5mm and insulation thickness was set to 0.5mm. Conductor 2 and conductor 1 lengths varied by simulation and the contact length was set to 1.5mm.

An insulated lead with a circular cross-section, consisting of two conductive segments with varying lengths, $L_{1}$ and $L_{2}$, was designed to evaluate the change in the maximum 1g-SAR at the lead’s tip. The lead had a diameter of 1.5 mm, an initial insulation thickness of 0.5 mm $\left(\varepsilon_{r}=2.5\right)$, and a total length of 40 cm. A platinum contact of 1.5 mm in length and a conductivity of 9.3×10^6^ S/m was placed at the tip of the lead in contact with conductor $L_{2}$, as illustrated in Figure 2.

The total resistance of the lead was set to 400 ohms, in line with the value chosen in previous research on the resistive tapered stripline [7]. The conductivity of each segment was set using Equations 1 and 2, incorporating the lengths of conductors 1 and 2, the conductivity ratio $\sigma_{1}/\sigma_{2}$ of the two segments, the lead diameter d, and the total resistance $R_{\mathrm{total}}$.


(1) $$\sigma_{1}=\frac{L_{1}+L_{2}\left(\sigma_{1}/\sigma_{2}\right)}{\pi (d/2{)}^{2}}\frac{1}{R_{\mathrm{total}}}$$

(2) $$\sigma_{2}=\frac{L_{1}+L_{2}\left(\sigma_{1}/\sigma_{2}\right)}{\pi (d/2{)}^{2}}\frac{1}{R_{\mathrm{total}}\left(\sigma_{1}/\sigma_{2}\right)}$$

The lead was placed 4cm away from the edge of the phantom, and a high-mesh resolution cubic area of 20mm×20mm×20mm around the tip of the lead to improve the accuracy of SAR calculations. The maximum 1g-averaged SAR was calculated using the built-in SAR module in HFSS software, in accordance with the recommendations of IEEE/IEC STD 62704–4 [10].

### C. Parametric Simulations

The three simulation variables evaluated for SAR reduction were the conductivity ratio ($\sigma_{1}/\sigma_{2}$), the phantom conductivity, and the insulation thickness. First, the conductivity ratio was set to either 1, 2, or 77. These values were chosen to match the conductivity ratios used in prior work [7]. Second, the conductivity of the phantom was varied from 0.1 S/m to 1.0 S/m. Lastly the insulation thickness was varied from 0.3mm to 0.7mm.

For the three parametric simulations the parametric variable of interest was set, and then solved with the $L_{2}$ value spanning the range from 10mm to 390mm. The $L_{1}$ value was adjusted to maintain a total wire length of 40cm. The maximum 1g-SAR near the tip of the lead was determined for all variations.

For the parametric evaluation of the conductivity ratio, a baseline SAR value was determined as the average of the SAR values when the conductivity ratio was set to 1. This was chosen to represent the expected SAR value if the multi-segment lead were replaced by a one-segment lead with a single conductivity throughout. To quantify the effects of each variation, for each simulation of $L_{2}$ from 10mm to 390mm the $L_{2}$ value of the minimum SAR value was determined.

## III. Results

### A. Conductivity Ratio

**Fig. 3.**
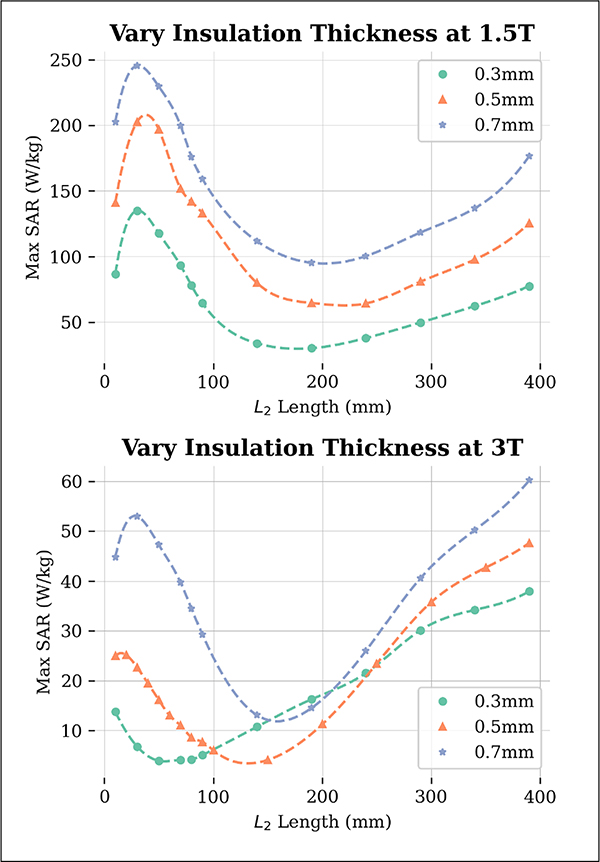
Maximum of 1g-SAR at the tip of the lead for various conductivity ratios of the two conductive lead segments at 64 MHz (top) and 127 MHz (bottom). The maximum SAR reduction was seen at a conductivity ratio of 77 at both field strengths. Dotted lines represent a spline fit to data points.

The simulation results showed that the largest SAR reduction was achieved when the conductivity ratio was set to 77 at both field strengths (see Figure 3). The baseline SAR values were 128 W/kg for 64 MHz and 50 W/kg for 127 MHz ($\sigma_{1}/\sigma_{2}=1$). Minimum SAR values were obtained with a conductivity ratio of 77 and $L_{2}$ length of 200 mm for 64 MHz ($\sigma_{1}=22069\;S/m$, $\sigma_{2}=287\;S/m$) and 140 mm for 127 MHz ($\sigma_{1}=15618\;S/m,\;\sigma_{2}=203\;S/m$).

The results also showed that the use of a conductivity ratio of 77 resulted in a 2-fold reduction in SAR at 64 MHz and an 11-fold reduction in SAR at 127 MHz when compared to baseline values. The results are summarized in Table I.

### B. Phantom Conductivity

**Fig. 4.**
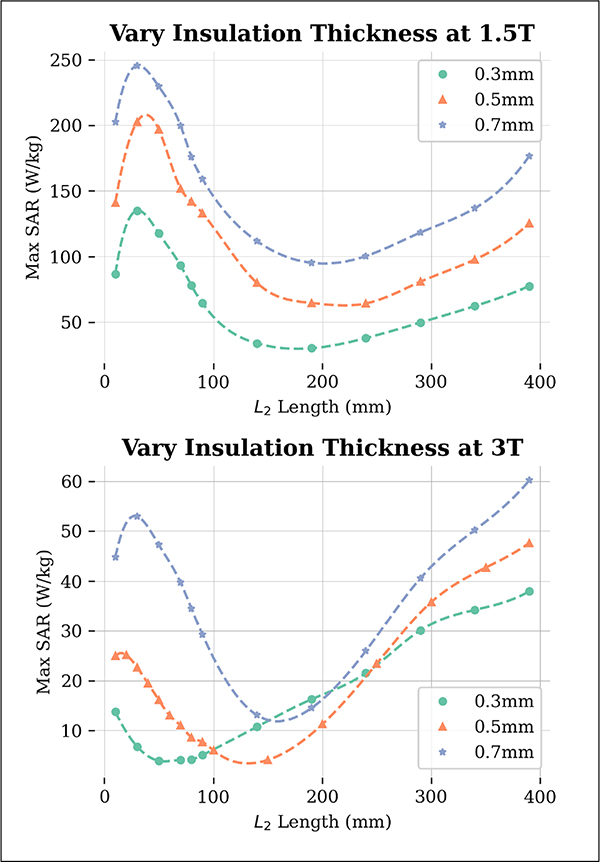
Maximum of 1g-SAR at the tip of the lead for varying phantom conductivities at 64 MHz (top) and 127 MHz (bottom). For all phantom conductivities, $\sigma_{1}/\sigma_{2}=77$. Dotted lines represent a spline fit to data points.

Figure 4 gives the maximum of 1g-SAR for various phantom conductivities. At 1.5 T, as the conductivity of the phantom increases, the 1g-SAR displays a continuous decrease for all $L_{2}$ values. The $L_{2}$ value that results in the minimum SAR remains unchanged when the phantom conductivity is altered. Conversely, at 3 T, the SAR reaches its highest point when the phantom conductivity is 0.47 S/m, with the SAR decreasing when phantom conductivity is either increased or decreased. The minimum SAR for each tested phantom conductivity is documented in Table II.

### C. Insulation Thickness

**Fig. 5.**
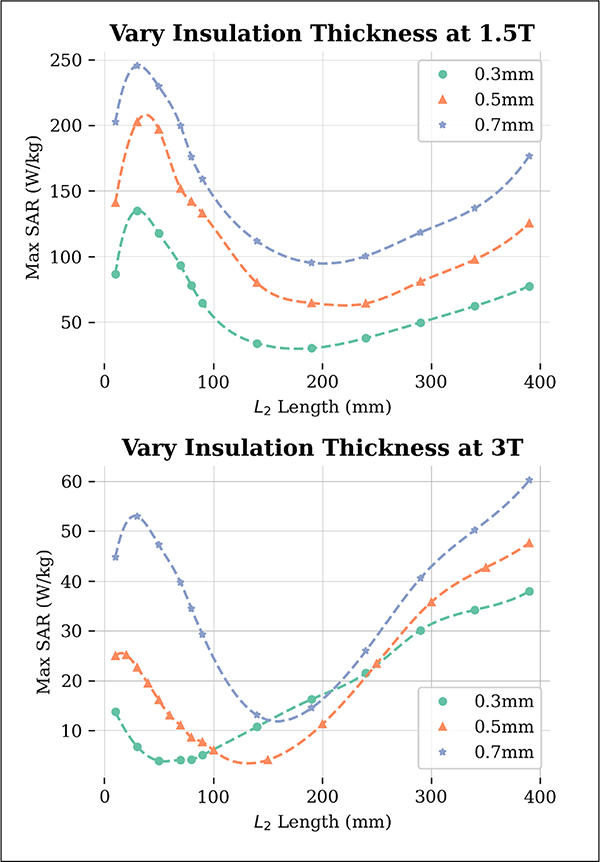
Maximum 1g-SAR results from varying the conductivity of the phantom medium for 64 MHz (top) and 127 MHz (bottom). The $L_{2}$ at which the SAR was reduced grew larger as the insulation thickness increased. The maximum SAR values also increased across all $L_{2}$ values as the insulation thickness increased. For all insulation thicknesses, $\sigma_{1}/\sigma_{2}=77$. Dotted lines represent a spline fit to data points.

The results of the effect of insulation thickness on SAR values are presented in Figure 5. It is evident that as the insulation thickness increases, the maximum 1g-SAR also increases for both 1.5 T and 3 T. The $L_{2}$ value at which the minimum SAR occurs increases with increasing insulation thickness, with a greater impact observed at 3 T. The minimum SAR values for each insulation thickness are given in Table III.

## IV. Discussion & Conclusions

Novel approaches for reducing RF induced heating during MRI continue to be developed and tested. These approaches include modified RF coils [11], the use of open bore scanners [12], [13] among others. Implementation of these techniques in a patient-facing way is limited as there are significant changes to existing MRI technology required.

In this study, we achieved large reductions in the SAR by using a two-segment cylindrical wire with differing conductivities at both 64 MHz and 127 MHz. This result is consistent with prior work on resistive striplines [8], [7] and expands their relevance to 1.5T MRI systems. Across all simulations, the 64 MHz maximum 1g-SAR values were larger than the 127 MHz values at the comparable values of $L_{2}$. This is consistent with an increased resonant effect seen at 64 MHz because the total lead length (40cm) is closer to the wavelength of the incident RF field [14]. Modification of the insulation thickness showed a strong effect on the $L_{2}$ value at the minimum SAR as well as the value of the minimum SAR at 127 MHz. Physical prototypes have been constructed by controlling the thickness of a thin-film vapor deposition of titanium and gold which demonstrate continued usability for stimulation therapy. [7]. In future prototype construction with a cylindrical lead model, the $L_{2}$ value to achieve the minimum SAR will be very sensitive to the insulation thickness and expected medium conductivity.

These preliminary findings demonstrate that it is possible to reduce the SAR at the tip of a long lead by using a two-segment lead construction. Further testing is required to assess the potential reduction in temperature as well as construction of physical leads for experimental testing.

## Figures and Tables

**TABLE I:** Minimum SAR values for all conductivity ratios evaluated. The conductivity ratio of 1 can be interpreted as the baseline SAR because the SAR near the lead tip is not expected to vary significantly as $L_{2}$ is varied when the conductivities of the two segments are identical.

Conductivity Ratio	64 MHz Min SAR (W/kg)	127 MHz Min SAR (W/kg)

1	119.1	48.0
2	92.8	25.0
77	64.3	4.1

**TABLE II:** Minimum SAR values for all phantom conductivities evaluated.

Phantom Conductivity	64 MHz Min SAR (W/kg)	127 MHz Min SAR (W/kg)

0.1 S/m	260.8	1.7
0.3 S/m	103.2	3.4
0.47 S/m	64.3	3.6
0.7 S/m	41.0	4.1
1.0 Sm/	26.0	4.1

**TABLE III:** Minimum SAR values for all insulation thicknesses evaluated.

Insulation Thickness (mm)	64 MHz Min SAR (W/kg)	127 MHz Min SAR (W/kg)

0.3	30.1	3.9
0.5	64.3	4.1
0.7	95.1	13.1

## Clinical relevance—

Design of an implantable segmented lead has potential to mitigate RF heating concerns and open a wider patient population to both 1.5T and 3T MRI scans.
